# Pain and quality of life in nursing home residents with dementia after admission – a longitudinal study

**DOI:** 10.1186/s12913-023-10041-5

**Published:** 2023-09-27

**Authors:** Anne-S. Helvik, Sverre Bergh, Jūratė Šaltytė Benth, Tom Borza, Bettina Husebø, Kjerstin Tevik

**Affiliations:** 1 Department of Public Health and Nursing, Faculty of Medicine and Health Sciences, Norwegian University of Science and Technology (NTNU), Trondheim, Norway Box 8905, NO-7491 Trondheim,; 2 Norwegian National Centre for Ageing and Health, Vestfold Hospital Trust, Tønsberg, Norway; 3 Research Centre for Age-Related Functional Decline and Disease, Innlandet Hospital Trust, Ottestad, Norway; 4 Institute for Clinical Medicine, University of Oslo, Oslo, Norway; 5 Centre for Elderly and Nursing Home Medicine, Department of Global Public Health and Primary Care, University of Bergen, Bergen, Norway; 6 Department of Global Public Health and Primary Care, Neuro-SysMed, University of Bergen, Bergen, Norway

**Keywords:** Behavioral and psychological symptoms, Elderly, Cognitive impairment, Drug use, Long term facilities, Neuropsychiatric symptoms, Older people, Pain medication, Quality indicator, Well-being

## Abstract

**Background:**

Pain in nursing home (NH) residents with dementia is commonly reported and may affect Quality of Life (QoL) negatively. Few longitudinal studies have explored how pain and QoL develop in NH residents with dementia starting from their admission to the NH.

**Aim:**

The aim was to explore pain, QoL, and the association between pain and QoL over time in persons with dementia admitted to a NH.

**Methods:**

A convenience sample, drawn from 68 non-profit NHs, included a total of 996 Norwegian NH residents with dementia (mean age 84.5 years, SD 7.6, 36.1% men) at NH admission (A_1_), with annual follow-ups for two years (A_2_ and A_3_). Pain and QoL were assessed using the Mobilization-Observation-Behavior-Intensity-Dementia-2 (MOBID-2) Pain Scale and the Quality of Life in Late-Stage Dementia (QUALID) scale, respectively, at all assessments. Severity of dementia, personal level of activities of daily living, general medical health, neuropsychiatric symptoms, and the prescription of psychotropic drugs and analgesics (opioids and/or paracetamol) were also assessed at all assessments.

**Results:**

Mean (SD) MOBID-2 pain intensity scores were 2.1 (2.1), 2.2 (2.2), and 2.4 (2.1) at A_1_, A_2_, and A_3_, respectively. Participants who were prescribed analgesics had higher pain intensity scores at all assessments than participants not prescribed analgesics. The mean (SD) QUALID scores at each assessment were 19.8 (7.1), 20.8 (7.2), and 22.1 (7.5) at A_1_, A_2_, and A_3_, respectively. In the adjusted linear mixed model, higher pain intensity score, prescription of opioids, and prescription of paracetamol were associated with poorer QoL (higher QUALID total score and higher scores in the QoL dimensions of sadness and tension) when assessed simultaneously. No time trend in QoL was found in these adjusted analyses.

**Conclusion:**

NH residents with dementia who have higher pain intensity scores or are prescribed analgesics are more likely to have poorer QoL. Clinicians, NH administrators, and national healthcare authorities need to look into strategies and actions for pharmacological and non-pharmacological pain treatment to reduce pain intensity while simultaneously avoiding negative side effects of pain treatment that hamper QoL.

**Supplementary Information:**

The online version contains supplementary material available at 10.1186/s12913-023-10041-5.

## Introduction

People admitted to nursing homes (NHs) usually have extensive care needs due to advanced age, several impairments, and dementia [1–10], and several have pain difficulties.

Internationally, studies have found the prevalence of pain in NH residents to be up to 80% [7, 11–14]. It has been reported that residents with severe dementia have pain more often than those with less severe dementia [15], but the findings are inconclusive [16–18]. In Norway, a cross-sectional study of NH residents with dementia reported that 43% of them had clinically relevant pain, independent of length of stay prior to assessment [19], while a recent study found that 35.5% of NH residents had clinically relevant pain shortly after their admission [20]. A Dutch study reported that 52% of NH residents with dementia had pain shortly after their admission to the NH [15]. To the best of our knowledge, few studies have reported on the severity of pain intensity in an observational longitudinal study in newly-admitted NH residents with dementia [15, 21]. NH residents with moderate or severe dementia may have reduced capacity to report their pain, either due to cognitive impairment and/or to language problems [20, 22, 23]. Typical pain behavior, such as verbalization/vocalization (e.g., sighing, moaning, calling out, and gasping), facial expressions (e.g., grimacing, and frowning), and defensive postures (e.g., freezing, tensing, guarding, pushing, and crouching), may be prominent signs of pain in people with dementia [24–27]; thus, staff may observe such actions using one of the many existing measures for discerning pain behavior [28]. The Mobilization-Observation-Behavior-Intensity-Dementia-2 (MOBID-2) Pain Scale and the Pain Assessment in Advanced Dementia (PAINAD) scale are the inventories most frequently used to assess pain in NH residents with dementia [18, 20, 29–33].

Systematic pain assessment is a prerequisite for adequate pain treatment, and adequate pain treatment is essential for older adults—both with and without dementia [24, 28]. However, pain treatment in older adults and NH residents may be complicated, due to aging and additional co-morbidities to dementia, and thus precautions should be taken [24, 28, 34]. Paracetamol is recommended as the first-line pharmacological treatment for pain in older adults [35]. At the recommended doses, it is regarded as relatively safe to prescribe paracetamol to NH residents [36] with and without dementia [34, 37], but knowledge about adverse effects and effects after long-term use of paracetamol in residents with dementia is limited [37]. Prescription of opioids has been recommended for treatment of moderate to severe pain intensity [35]. However, a careful risk–benefit analysis is necessary for each individual [34], since side effects of anticholinergics may provoke considerable adverse events in people with dementia [34, 38].

Undiagnosed, untreated, or partly-treated pain in NH residents may trigger neuropsychiatric symptoms such as aggression, psychosis, affective symptoms, and apathy [27, 39]. Furthermore, cross-sectional NH studies have reported that pain and pain intensity are associated with poorer Quality of Life (QoL) in residents with dementia [20, 40–43]. However, the results diverge when it comes to the association between prescribed analgesics and QoL: some cross-sectional studies find analgesics independently associated with poorer QoL [44], while other have not found such an association [19, 20]. A systematic review regarding QoL that included 10 cross-sectional studies published before 2010 found depression, neuropsychiatric symptoms, impairment in activities of daily living (ADL), more severe cognitive impairment, and use of psychotropic drugs to be associated with poorer QoL [45]. However, to the best of our knowledge, few studies have explored the association between pain and QoL in NH residents using a longitudinal design and adjusting for health conditions and prescription of analgesics and psychotropic drugs.

There are several conceptual frameworks and definitions of QoL, but no single, clear, and universally accepted definition exists, neither universally [46] nor for persons with dementia [47, 48]. Consequently, the variety of dementia-specific QoL inventories is extensive [47], although several authors have highlighted the need for a QoL inventory that emphasizes psychological status as well as participation, comfort, and/or joy in activities [47–52]. Furthermore, it is important to capture QoL in all individuals with dementia, regardless of the severity of their condition; thus, a proxy-based inventory may be appropriate in studies of QoL in NH residents with severe dementia and reduced ability to express their QoL. Consequently, the Quality of Life in Late-Stage Dementia (QUALID) scale [53] was chosen for the study.

In this large-scale longitudinal study, the first aim was to describe the pain intensity and QoL of persons with dementia at admission to a NH and after 12 and 24 months. The second aim was to explore the association between pain intensity and QoL in the same residents with dementia over time, adjusting for prescription of analgesics, psychotropic drugs, and several health conditions assessed simultaneously.

## Methods

### Design

This is an observational longitudinal study of NH residents, who were followed from their admission for up to two years, in a convenience sample drawn from 68 non-profit NHs operated and owned by 32 municipalities in southeastern Norway. The NHs were located in rural and urban areas of one county. The baseline data was collected from November 2014 to December 2019, and the follow-up data were collected annually or until the participant’s death. The final follow-up data collection was completed in December 2021.

### Participation and setting

The jurisdiction that provides NH care and treatment around the clock in Norway is the local municipalities [54]. Approximately 40,000 NH places (beds) are available for a population of about 5.5 million [55].

In 2014–2019, a total of 3,318 residents were registered as being admitted to one of the 68 public NHs participating in our study [21]. For the present study, 1,283 residents with an expected stay of longer than four weeks were recruited. All residents 65 years and older, independent of whether they had established dementia or not, and residents younger than 65 years with established dementia were asked to participate. The only exclusion criterion was a life expectancy of less than six weeks. The residents who did not participate (*n* = 2,035)—due to death shortly after admission (*n* = 238), not accepting the invitation (*n* = 567), or other unknown reasons (*n* = 1,230)—were more often women but they did not differ in age from those recruited (i.e., mean (SD) age 84.2 (8.6) and 84.5 (7.6) years, respectively) [21].

**Fig. 1:**
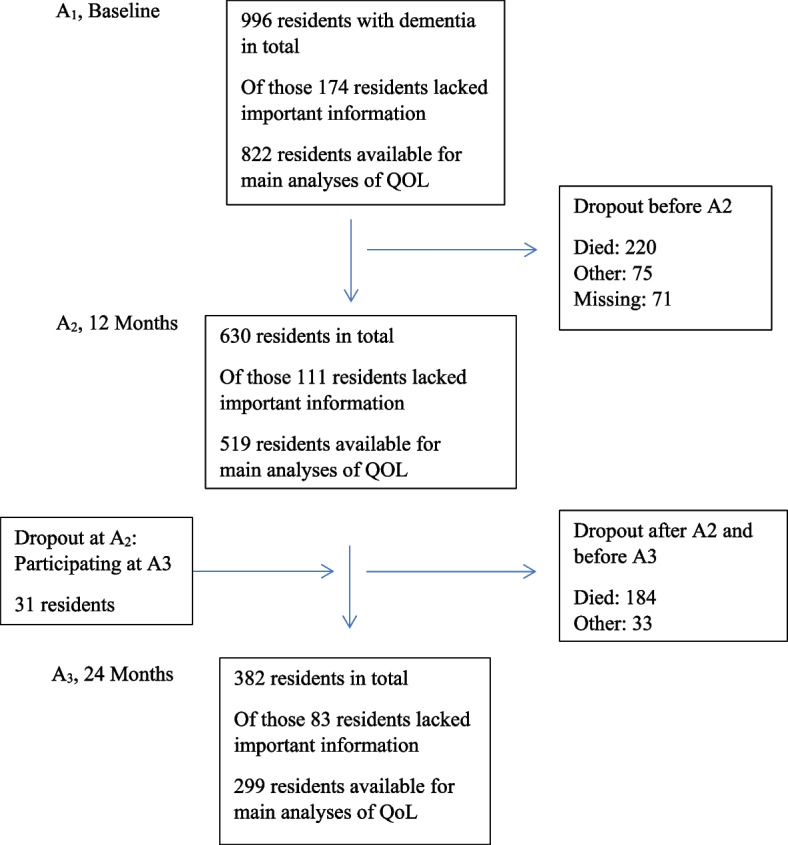
Flowchart

The present study included only people with dementia at admission. Two physicians with extensive experience in research and clinical old age psychiatry independently diagnosed dementia at admission according to the ICD-10 criteria, based on all available information. A third physician, also with extensive experience, was consulted in situations where the two physicians disagreed. In total, 1,074 residents had dementia, 201 did not have dementia, and eight could not be diagnosed. Of those with dementia, 78 residents lacked information about pain severity. Consequently, the present study included 996 residents with dementia admitted to a NH (baseline, A_1_). The numbers of participants assessed at 12 months (A_2_) and at 24 months (A_3_) were 630 and 299, respectively. Due to missing information, the numbers of residents suitable for the most complex analyses were 822 at A_1_, 519 at A_2_, and 299 at A_3_ (Fig. 1). Mean (SD) duration from A_1_–A_2_ and from A_2_–A_3_ was 341 (82.7) and 356 (66.9) days, respectively; mean (SD) time of follow-up was 683 (98.2) days.

### Measures

QoL was assessed at all assessments using the QUALID scale, a brief proxy-based inventory that includes both positively and negatively worded items [53]. The QUALID scale rates 11 different observable behaviors on a 5-point Likert scale (1-5), with higher sum scores indicating poorer QoL (score range 11–55). The inventory is administered as a structured interview with an informant who knows the person with dementia—a healthcare professional who had spent a considerable part of at least three of the previous seven days with the person [53]. In this study, the nursing personnel who knew the resident best answered the interview. Previous principal component analyses have shown that three dimensions of QoL—tension (including being physically uncomfortable, verbalization that suggests discomfort, irritability, and appearing calm), sadness (including crying, appearing sad, and facial expression of discomfort), and well-being (including smiles, enjoying eating, enjoying social interaction, and enjoying touching/being touched)—explained 53% of the variance [56]. The QUALID scale has been translated to Norwegian and validated in several samples of NH residents with dementia [56–58].

The MOBID-2 Pain Scale, applied at all assessments, is an observational pain tool for people with dementia [23, 59]. The MOBID-2 scale measures nociceptive, musculoskeletal pain during active, guided movements as well as pain that might be related to the internal organs, head, and skin during the previous week, documented on a body chart to show potential pain location. The scale consists of 10 items, wherein each item’s score ranges from 0 to 10, with a higher score indicating more severe pain; the sum score of these 10 items ranges from 0 (least severe) to 100 (most severe). In addition, a separate item grades the overall pain intensity from 0 to 10 (most severe pain intensity); an overall score of ≥ 3 indicates that the resident has clinically relevant pain intensity [23]. In this study, the nursing personnel who knew the resident best answered the scale. The scale has been psychometrically tested for its validity, reliability, and responsiveness and has been used in several studies among NH residents, including in Norway [59–61].

Data on prescribed medications were collected from each resident’s medical record at all assessments. Only medication prescribed for regular use was recorded in this study. The prescriptions were sorted according to the Anatomical Therapeutic Chemical (ATC) classification system [62]. ATC codes beginning with N02 were divided into opioids (N02A) and paracetamol (N02B E01, N02A J06, and N02A J13). Psychotropic drugs were categorized into antipsychotics (N05A except lithium), antidepressants (N06A), anxiolytics (N05B), and hypnotics/sedatives (N05C) [62]. Use of opioids, paracetamol, and any analgesic were dichotomized into yes or no. The number of prescribed psychotropic drugs were categorized as 0, 1, 2, and ≥ 3.

Neuropsychiatric symptoms (NPS) were measured at all assessments using the Neuropsychiatric Inventory Nursing Home version (NPI-NH) [63]. The 12-item inventory, scored by the nursing personnel who knew the resident best, includes severity (scores 1–3) and frequency (scores 1–4) of the following symptoms: delusion, hallucination, euphoria, agitation/aggression, disinhibition, irritability/lability, depression/dysphoria, anxiety, apathy/indifference, aberrant motor behavior, night-time behavior disturbances, and appetite and eating disorders (yes/no). Each symptom, if it exists, is scored from 1 to 12—i.e., the product of the severity score and frequency score. Three sub-syndromes have been established based on a factor analysis: agitation (including agitation/aggression, disinhibition, and irritability), affective (including depression and anxiety), and psychosis (including delusions and hallucination) [64]. The NPI-NH has been translated to Norwegian and validated [65].

The severity of dementia was scored at all assessments using the Clinical Dementia Rating (CDR) scale by the nursing personnel who knew the resident best. The CDR scale covers six domains—memory, orientation, judgment and problem solving, community affairs, home and hobbies, and personal care [66]—with five response options (0, 0.5, 1, 2, 3) for each domain [66, 67]. The categorical end score (0, 0.5, 1, 2, or 3) is calculated using an algorithm that gives priority to memory [66, 67]. The categories indicate level of dementia, ranging from 0 (no dementia) to 3 (severe dementia). The present study also used a sum-score of the six domains (CDR Sum of Boxes, CDR-SoB); this score ranged from 0 to 18 with a higher score indicating more severe dementia [68–70]. The correlation between the categorical CDR and the CDR-SoB is high [71, 72]. The CDR scale has been translated to Norwegian and used in several NH studies [8, 60].

The Physical Self-Maintenance Scale (PSMS) [73] was used at all assessments to assess personal activities of daily living (P-ADL). The scale includes six items, with a total score range of 6–30, with higher scores indicating a lower level of functioning [73]. The nursing personnel who knew the resident best completed the scale. This scale is frequently used in Norwegian NH studies [68, 74].

The General Medical Health Rating (GMHR) scale was used at all assessments to assess physical health [75]. This is a one-item global rating scale with four response options: good, fairly good, fairly poor, and poor. The rating was based on all available information of physical health and use of prescribed medication, and it was performed by the nursing personnel who knew the resident best. The scale has previously been used in large NH studies, including those on older people with dementia in Norway [68].

Demographic information (age, gender, and marital/partner status) was collected from medical records, and civil status was checked at all follow-ups. The type of NH unit in which the resident lived was categorized at their admission either as a regular unit or a special care unit for people with dementia.

### Procedure

Data were collected by healthcare professionals at the NHs and supervised by 10 research nurses. Data collectors were primarily registered nurses (74%) who had completed a two-day training program prior to the data collection. All baseline (A_1_) information on residents was collected over the first month of their NH stay by a standardized interview with the residents, their next of kin, and their caregivers in the NH, along with a review of their medical records.

The NH staff, including the NH physician, assessed the residents’ capacity to consent to participate in the study. All residents who had the capacity to do so gave their written consent. If a resident had limited capacity to consent, the resident’s next of kin consented on behalf of the resident. These procedures have been recommended and approved by the Norwegian Regional Ethics Committee South East (2014/917).

### Statistics

Baseline characteristics were described in means and standard deviations (SDs) for continuous variables and in frequencies and percentages for categorical variables. Data were collected at different NHs implying a hierarchical structure: thus, residents with clinically relevant pain (overall MOBID-2 ≥ 3) and residents without clinically relevant pain (overall MOBID-2 < 3) at A_1_ were compared by generalized linear mixed model with random intercepts for NHs. The same model was used to compare residents prescribed and not prescribed analgesics with respect to single MOBID-2 items, overall MOBID-2 scores, summed QUALID items (overall QoL), three QUALID (QoL) dimensions, and single QUALID items.

Factors associated with overall QoL and the three dimensions of QoL were assessed by linear mixed model with random effects for patients nested within NHs. First the model was estimated with fixed effects for time (coded as dummies). Then, pre-chosen factors assessed simultaneously with QUALID (overall QoL and dimensions of QoL) were entered into the model one by one. Finally, a multiple model containing time and all factors was estimated. The pre-chosen factors were MOBID-2 sum-score and prescription of analgesics (opioids yes/no, paracetamol yes/no) at all assessments, as well as severity of dementia, physical health (poor/fairly poor versus good/fairly good), P-ADL functioning (PSMS), NPI-NH subsyndromes (agitation, affective, and psychosis) and NPI-NH symptom absence, number of prescribed psychotropic drugs (0, 1, 2, ≥ 3), and civil status (married/partner or not) at all assessments, and age, gender, and NH unit (regular care unit or dementia care unit) at baseline.

Only cases with no missing values on covariates were included in the regression analyses. All tests were two-sided, and results with *p*-values below 0.05 were considered significant. The statistical analyses were performed in SPSS version 27 and STATA version 17.

## Results

The mean (SD) age of the 996 admitted NH residents with dementia who were included in the study was 84.5 (7.6) years, and 360 (36.1%) of them were men. The mean (SD) CDR-SoB score was 11.2 (3.5). Compared to those without clinically relevant pain (MOBID-2 < 3), residents with clinically relevant pain (MOBID-2 ≥ 3, 35.6%) more often had poor physical health, higher PSMS scores (poorer P-ADL functioning), and higher NPI scores of agitation, affective, and psychosis subsyndromes; they also more often used any psychotropic drugs (Table 1).

**Table 1:** Sample characteristics of newly admitted NH residents with dementia by clinically relevant pain^1^

Characteristics	MOBID-2 ≥ 3 *n* = 355 (35.6%)	MOBID-2 < 3 *n* = 641 (64.3%)	*p*-value^1^
*Socio-demographics*			
Age, mean (SD)	84.3 (7.6)	84.7 (7.6)	0.502
Males, n (%)	118 (33.2)	242 (37.8)	0.141
Married/partner, n (%) (11 missing)	118 (33.5)	192 (30.3)	0.274
*Health condition*			
CDR-SoB, mean (SD) (41 missing)	11.4 (3.4)	11.1 (3.5)	0.234
GMHR, n (%) (65 missing)			
Fairly poor/Poor	204 (60.7)	276 (46.4)	< 0.001
Good/Fairly good	132 (39.3)	319 (53.6)	
PSMS score, mean (SD) (4 missing)	15.5 (4.5)	14.7 (4.5)	0.002
NPI-NH sub-syndrome^a^			
Agitation, mean (SD) (30 missing)	5.8 (7.8)	4.4 (7.4)	0.008
Affective, mean (SD) (33 missing)	4.4 (5.9)	3.0 (4.9)	<0.001
Psychosis, mean (SD) (21 missing)	2.5 (4.7)	1.7 (3.7)	0.004
Apathy, mean (SD) (21 missing)	1.1 (2.5)	1.0 (2.4)	0.346
Psychotropic drugs (yes), n (%)			
Antipsychotics	43 (12.1)	71 (11.1)	0.614
Antidepressants	127 (35.8)	175 (27.3)	0.012
Anxiolytics	55 (15.5)	85 (13.3)	0.377
Sedatives	107 (30.1)	144 (22.5)	0.009
Any	214 (60.3)	335 (52.3)	0.021
Analgesics, n (%)			
Opioids	107 (30.1)	73 (11.4)	< 0.001
Paracetamol	213 (60.0)	240 (37.4)	< 0.001
Any	237 (66.8)	258 (40.3)	< 0.001
MOBID-2, sum score^b^, mean (SD)	16.4 (10.2)	3.5 (4.4)	< 0.001
*NH characteristics*, n (%) (18 missing)			
Regular care unit	209 (60.2)	355 (56.3)	0.285
Special care unit	138 (39.8)	276 (43.7)	
*Type of dementia*, n (%)			
Alzheimer’s disease	222 (62.5)	393 (61.3)	0.285
Vascular dementia	32 (9.0)	35 (5.5)	
Alzheimer’s disease mixed type	36 (10.1)	72 (11.2)	
Frontotemporal dementia	28 (7.9)	66 (10.3)	
Lewy body dementia/ Parkinson’s disease	31 (8.7)	55 (8.6)	
Unspecified	6 (1.7)	20 (3.1)	

Abbreviations: *CDR-SoB* Clinical Dementia Rating-Sum of Boxes, *GMHR* General Medical Health Rating, *MOBID-2* Mobilization-Observation-Behavior-Intensity-Dementia-2, *n* number, *NH* Nursing Home, *NPI-NH* Neuropsychiatric Inventory Nursing Home version, *Opioids* N02A, *Paracetamol* N02B E01 N02A J06 and N02A J13, *PSMS* Physical Self-Maintenance Scale, *SD* Standard Deviation

Clinically relevant pain: Overall pain intensity with MOBID-2 ≥ 3

^1^Generalized linear mixed model (adjusting for cluster effect within NH)

^a^NPI-NH Agitation sub-syndrome: agitation/aggression, disinhibition, and irritability, NPI-NH Affective sub-syndrome: depression and anxiety, NPI-NH Psychosis sub-syndrome: delusions and hallucination

^b^The sum score from the ten single MOBID-2 items

### Pain intensity and degree of quality of life

The mean (SD) overall MOBID-2 pain intensity scores were 2.1 (2.1), 2.2 (2.2), and 2.4 (2.1) at baseline (A_1_), A_2_, and A_3_, respectively. For a description of single-item scores, see Table 2. At all assessments, residents who were prescribed analgesics had a higher overall pain intensity compared to residents who were not prescribed analgesics. The pain intensity scores of all single areas of pain were higher at all assessments for those prescribed analgesics, except for head/mouth/neck at A_1_ and A_3_ and skin at A_3_.

**Table 2:** Pain assessed with MOBID-2 and quality of life assessed with QUALID by prescribed analgesics^a^

	**A1**		**A2**		**A3**	
	All (*N* = 996)	Analgesics^a^ Yes/No (*N* = 495/501)	All (*N* = 630)	Analgesics^a^ Yes/No (*N* = 410/220)	All (*N* = 382)	Analgesics^a^ Yes/No (*N* = 272/110)
	Mean (SD)	Mean (SD)	Mean (SD)	Mean (SD)	Mean (SD)	Mean (SD)
*Pain assessed with MOBID-2*						
Mobilization one day						
Opening hands (0–10)	0.40 (1.22)	0.54 (1.39)/0.26 (1.00)*	0.44 (1.29)	0.54 (1.46)/0.25 (0.87)***	0.55 (1.50)	0.67 (1.63)/0.26 (1.06)**
Stretch arms (0–10)	0.99 (1.95)	1.37 (2.25)/0.61 (1.50)*	1.12 (2.06)	1.34 (2.21)/0.72 (1.70)*	1.15 (2.09)	1.36 (2.24)/0.63 (1.52)***
Bend and stretch knees and hips (0–10)	1.32 (2.17)	1.82 (2.55)/0.83 (1.58)*	1.44 (2.24)	1.83 (2.42)/0.72 (1.60)*	1.60 (2.35)	1.86 (2.53)/0.95 (1.68)***
Turn in bed (0–10)	1.15 (2.03)	1.67 (2.38)/0.65 (1.45)*	1.32 (2.19)	1.74 (2.45)/0.52 (1.24)*	1.38 (2.19)	1.61 (2.34)/0.79 (1.59)***
Sit bedside (0–10)	1.13 (2.02)	1.70 (2.41)/0.57 (1.31)*	1.31 (2.13)	1.68 (2.31)/0.62 (1.49)*	1.33 (2.12)	1.64 (2.32)/0.57 (1.19)*
Observations last week						
Head, mouth, neck (0–10)	0.63 (1.61)	0.72 (1.78)/0.54 (1.41)	0.70 (1.77)	0.93 (2.06)/0.25 (0.84)*	0.61 (1.44)	0.65 (1.49)/0.50 (1.31)
Heart, lung, chest wall (0–10)	0.41 (1.32)	0.51 (1.48)/0.30 (1.13)**	0.52 (1.56)	0.62 (1.65)/0.33 (1.35)**	0.44 (1.37)	0.55 (1.54)/0.18 (0.73)**
Abdomen (0–10)	0.38 (1.19)	0.47 (1.29)/0.29 (1.08)**	0.49 (1.40)	0.59 (1.52)/0.32 (1.13)**	0.63 (1.50)	0.68 (1.56)/0.49 (1.33)
Pelvis, genital organs (0–10)	1.05 (1.97)	1.38 (2.23)/0.74 (1.62)*	1.18 (2.03)	1.57 (2.27)/0.45 (1.19)*	1.28 (2.12)	1.54 (2.28)/0.65 (1.45)*
Skin (0–10)	0.62 (1.48)	0.74 (1.63)/0.50 (1.31)**	0.73 (1.68)	0.88 (1.90)/0.44 (1.14)***	0.84 (1.73)	0.90 (1.78)/0.69 (1.58)
MOBID-2 overall evaluation score (range 0–10)	2.05 (2.13)	2.69 (2.29)/1.43 (1.75)*	2.21 (2.16)	2.79 (2.22)/1.10 (1.51)*	2.40 (2.08)	2.78 (2.15)/1.46 (1.55)*
*Quality of life*						
QUALID total score (11–55)	19.77 (7.14)	20.91 (7.72)/18.65 (6.32)*	20.83 (7.21)	21.86 (7.34)/18.88 (6.56)*	22.05 (7.52)	22.47 (7.44)/21.00 (7.64)
Dimensions						
Well-being dimension (4–20)	6.85 (2.44)	7.16 (2.69)/6.55 (2.12)*	7.32 (2.60)	7.49 (2.64)/6.99 (2.50)**	7.85 (2.84)	7.83 (2.79)/7.88 (2.97)
Sadness dimension (3–15)	5.73 (2.91)	6.02 (3.02)/5.43 (2.77)***	5.96 (2.99)	6.34 (3.10)/5.26 (2.63)*	6.11 (2.86)	6.25 (2.89)/5.79 (2.77)
Tension dimension (4–20)	7.20 (3.59)	7.73 (3.89)/6.67 (3.19)*	7.55 (3.72)	8.03 (3.82)/6.64 (3.35)*	8.09 (3.86)	8.38 (3.88)/7.36 (3.70)***
Single items						
Smile (1–5)	1.44 (0.89)	1.50 (0.96)/1.38 (0.81)**	1.53 (0.98)	1.56 (1.00)/1.46 (0.94)	1.70 (1.10)	1.67 (1.07)/1.76 (1.16)
Appears sad (1–5)	2.19 (1.45)	2.28 (1.52)/2.10 (1.37)**	2.25 (1.48)	2.37 (1.53)/2.02 (1.35)***	2.35 (1.51)	2.36 (1.51)/2.31 (1.52)
Cries (1–5)	1.48 (0.99)	1.52 (1.06)/1.43 (0.92)	1.54 (1.10)	1.62 (1.19)/1.40 (0.89)**	1.47 (1.00)	1.52 (1.08)/1.36 (0.76)
Has facial expressions of discomfort (1–5)	2.06 (1.15)	2.22 (1.17)/1.90 (1.10)*	2.17 (1.11)	2.34 (1.11)/1.84 (1.05)*	2.29 (1.12)	2.37 (1.08)/2.09 (1.20)**
Appears physically uncomfortable (1–5)	1.75 (1.09)	1.97 (1.16)/1.53 (0.97)*	1.86 (1.10)	2.02 (1.13)/1.55 (0.98)*	1.93 (1.05)	2.04 (1.07)/1.66 (0.96)*
Verbalizations suggests discomfort (1–5)	1.89 (1.35)	2.11 (1.47)/1.67 (1.19)*	2.01 (1.41)	2.17 (1.50)/1.69 (1.17)*	2.26 (1.54)	2.39 (1.56)/1.95 (1.43)**
Is irritable or aggressive (1–5)	1.68 (1.15)	1.77 (1.22)/1.60 (1.07)**	1.83 (1.23)	1.90 (1.27)/1.69 (1.12)**	1.97 (1.30)	1.99 (1.31)/1.91 (1.26)
Enjoys eating (1–5)	1.42 (0.93)	1.54 (1.07)/1.30 (0.76)*	1.56 (1.04)	1.62 (1.09)/1.45 (0.93)**	1.70 (1.15)	1.72 (1.15)/1.65 (1.15)
Enjoys touching and being touched (1–5)	2.20 (0.88)	2.25 (0.88)/2.16 (0.88)**	2.27 (0.90)	2.28 (0.89)/2.24 (0.93)	2.34 (0.89)	2.31 (0.88)/2.44 (0.92)
Enjoys interacting with others (1–5)	1.79 (0.90)	1.87 (0.94)/1.71 (0.85)***	1.96 (0.97)	2.03 (0.98)/1.83 (0.94)**	2.11 (0.99)	2.13 (0.98)/2.04 (1.01)
Appears calm and comfortable (1–5)	1.88 (1.13)	1.89 (1.14)/1.86 (1.11)	1.86 (1.12)	1.93 (1.13)/1.71 (1.09)**	1.93 (1.09)	1.96 (1.10)/1.84 (1.04)

^*^p < 0.001 for linear mixed model

^**^p < 0.05 for linear mixed model

^***^p < 0.01 for linear mixed model

*MOBID-2* Mobilization-Observation-Behavior-Intensity-Dementia-2

^a^Analgesics  paracetamol (N02B E01, N02A J06, and N02A J13) and/or opioids (N02A)

*N* = 996 at A1, but due to missing information it varies between 990–993 in the single items

QUALID Quality of Life of Late Stage Dementia

QUALID well-being dimension includes: smiles, enjoys eating, enjoys social interaction, and enjoys touching/being touched;

QUALID sadness dimension includes: cries, appears sad, and facial expression of discomfort;

QUALID tension dimension includes: physically uncomfortable, verbalization suggests discomfort, irritable, and appears calm

The overall mean (SD) QUALID score at baseline and the two follow-up assessments were 19.8 (7.1), 20.8 (7.2), and 22.1 (7.5), respectively (Table 2). The overall QUALID score, as well as the scores of the three dimensions of QUALID, were higher in residents prescribed analgesics than in residents not prescribed analgesics at A_1_ and A_2_, which indicates poorer QoL. At A_3_, only the score of the tension dimension of QUALID was higher in those prescribed analgesics.

### Factors associated with quality of life

In the unadjusted linear mixed model, there was a significant increase in overall QUALID score (i.e., poorer QoL) (Table 3) and in scores of separate dimensions of QoL over time, but in the adjusted analysis, no such trend was present (Table 4 and STable 1). In the adjusted linear mixed model of residents with dementia, higher MOBID-2 sum score (more severe pain intensity) was associated with higher QUALID total score (poorer overall QoL) when assessed simultaneously (Table 3). In an adjusted model among factors associated with the separate dimensions of the QUALID scale—i.e., sadness, well-being, and tension—higher pain intensity was associated with higher scores of the QUALID dimensions of sadness and tension, but not well-being, when assessed simultaneously (Table 4).

**Table 3:** Results of linear mixed model assessing factors associated with overall quality of life, QUALID total-score^a^

	Unadjusted models		Adjusted model	
	RC (95% CI)	*p*-value	RC (95% CI)	*p*-value
Time (months)				
0	0		0	
12	1.29 (0.69; 1.89)	** < 0.001**	0.02 (-0.44; 0.49)	0.919
24	2.50 (1.76; 3.25)	** < 0.001**	0.13 (-0.47; 0.73)	0.678
*Assessed simultaneously with outcome*				
MOBID-2	0.17 (0.14; 0.21)	** < 0.001**	0.08 (0.06; 0.10)	** < 0.001**
CDR-SoB	0.64 (0.54; 0.74)	** < 0.001**	0.17 (0.08; 0.25)	** < 0.001**
GMHR				
Poor/Fairly Poor – ref	0		0	
Good/Fairly good	-1.97 (-2.63; -1.31)	** < 0.001**	-0.50 (-0.98; -0.03)	**0.038**
PSMS	0.48 (0.41; 0.56)	** < 0.001**	0.20 (0.14; 0.27)	** < 0.001**
NPI-NH sub-syndrome^b^				
Agitation	0.47 (0.43; 0.50)	** < 0.001**	0.22 (0.19; 0.26)	** < 0.001**
Affective	0.81 (0.75; 0.86)	** < 0.001**	0.56 (0.51; 0.61)	** < 0.001**
Psychosis	0.60 (0.53; 0.68)	** < 0.001**	0.06 (-0.002; 0.13)	0.056
Apathy	0.78 (0.65; 0.91)	** < 0.001**	0.33 (0.23; 0.42)	** < 0.001**
Psychotropic drugs (number)				
0 – ref	0		0	
1	1.03 (0.25; 1.81)	**0.009**	0.24 (-0.30; 0.79)	0.380
2	2.77 (1.84; 3.71)	** < 0.001**	0.70 (0.04; 1.36)	**0.039**
3 +	5.30 (4.04; 6.57)	** < 0.001**	1.17 (0.26; 2.07)	**0.011**
Analgesics				
Opioids^c^	2.14 (1.30; 2.98)	** < 0.001**	0.69 (0.09; 1.30)	**0.025**
Paracetamol^d^	1.87 (1.19; 2.55)	** < 0.001**	0.61 (0.12; 1.10)	**0.015**
Civil status				
Unmarried/no partner – ref	0		0	
Married/partner	1.76 (0.90; 2.63)	** < 0.001**	0.37 (-0.22; 0.95)	0.217
*Assessed at baseline*				
Age	-0.07 (-0.12; -0.01)	**0.015**	0.008 (-0.03; 0.04)	0.666
Gender				
Females – ref	0		0	
Males	-0.29 (-1.15; 0.57)	0.509	-0.42 (-0.97; 0.14)	0.145
NH				
Regular care unit – ref	0		0	
Special care unit	0.65 (-0.19; 1.48)	0.129	-0.31 (-0.86; 0.24)	09.268

Bold values shown statistically significant result with a *p*-value less than 0.05

Abbreviations: *CI* Confidence interval, *CDR-SoB* Clinical Dementia Rating-Sum of Boxes, *GMHR* General Medical Health Rating, *MOBID-2* Mobilization-Observation-Behavior-Intensity-Dementia-2, *NH* Nursing Home, *NPI-NH* Neuropsychiatric Inventory Nursing Home version, *PSMS* Physical Self- Maintenance Scale, *RC* Regression coefficient, *QUALID* Quality of Life of Late Stage Dementia

^a^All analyses adjusted for cluster effect within NH; Only cases with no missing values on adjustment variables are included in the analyses

*N* = 822 (A_1_) + 519 (A_2_) + 299 (A_3_) = 1640

^b^NPI-NH Agitation sub-syndrome: agitation/aggression, disinhibition, and irritability; NPI-NH Affective sub-syndrome: depression and anxiety; NPI-NH Psychosis sub-syndrome: delusions and hallucination

^c^Opioids  N02A

^d^Paracetamol  N02B E01, N02A J06, and N02A J13

**Table 4:** Results of linear mixed model assessing factors associated with the three dimensions of quality of life: QUALID sadness, tension, and well-being ^a^

	**QUALID sadness **Adjusted model		**QUALID tension **Adjusted model		**QUALID well-being **Adjusted model	
	RC (95% CI)	*p*-value	RC (95% CI)	*p*-value	RC (95% CI)	*p*-value
Time (months)						
0	0		0		0	
12	0.002 (-0.22; 0.22)	0.985	-0.15 (-0.39; 0.10)	0.240	0.16 (-0.04; 0.37)	0.123
24	-0.09 (-0.37; 0.19)	0.542	-0.01 (-0.33; 0.30)	0.932	0.23 (-0.04; 0.49)	0.100
*Assessed simultaneously with outcome*						
MOBID-2	0.03 (0.02; 0.04)	** < 0.001**	0.05 (0.03; 0.06)	** < 0.001**	0.004 (-0.007; 0.02)	0.440
CDR-SoB	0.05 (0.01; 0.10)	**0.008**	0.08 (0.03; 0.12)	**0.001**	0.04 (-0.004; 0.08)	0.074
GMHR						
Poor/Fairly poor– ref	0		0		0	
Good/Fairly good	-0.09 (-0.31; 0.13)	0.426	-0.15 (-0.39; 0.09)	0.218	-0.26 (-0.48; -0.04)	**0.022**
PSMS	0.03 (-0.003; 0.06)	0.077	0.07 (0.03; 0.10)	** < 0.001**	0.11 (0.08; 0.14)	** < 0.001**
NPI-NH sub-syndrome^b^						
Agitation	0.007 (-0.01; 0.02)	0.433	0.20 (0.18; 0.22)	** < 0.001**	0.02 (-0.001; 0.03)	0.066
Affective	0.29 (0.27; 0.32)	** < 0.001**	0.21 (0.18; 0.24)	** < 0.001**	0.06 (0.04; 0.08)	** < 0.001**
Psychosis	0.01 (-0.02; 0.04)	0.445	0.05 (0.01; 0.08)	**0.005**	0.001 (-0.03; 0.03)	0.925
Apathy	0.08 (0.03; 0.12)	**0.001**	-0.02 (-0.07; 0.03)	0.346	0.26 (0.22; 0.31)	** < 0.001**
Psychotropic drugs (number)						
0 – ref	0		0		0	
1	0.20 (-0.05; 0.46)	0.117	0.09 (-0.19; 0.36)	0.547	-0.03 (-0.28; 0.22)	0.830
2	0.39 (0.08; 0.70)	**0.013**	0.23 (-0.11; 0.56)	0.183	0.14 (-0.17; 0.45)	0.382
3+	0.46 (0.04; 0.88)	**0.030**	0.42 (-0.03; 0.87)	0.070	0.26 (-0.17; 0.68)	0.235
Analgesics						
Opioids^c^	0.14 (-0.14; 0.42)	0.339	0.39 (0.08; 0.70)	**0.013**	0.18 (-0.10; 0.46)	0.211
Paracetamol^d^	0.25 (0.02; 0.48)	**0.035**	0.36 (0.11; 0.61)	**0.005**	0.02 (-0.21; 0.25)	0.852
Civil status						
Unmarried/no partner – ref	0		0		0	
Married/partner	0.14 (-0.14; 0.41)	0.328	0.24 (-0.05; 0.53)	0.108	0.009 (-0.27; 0.29)	0.950
*Assessed at baseline*						
Age	-0.0005 (-0.02; 0.02)	0.953	0.0007 (-0.02; 0.02)	0.936	0.008 (-0.01; 0.02)	0.392
Gender						
Females – ref	0		0		0	
Males	-0.42 (0.68; -0.17)	**0.001**	-0.12 )-0.39; 0.16)	0.404	0.14 (-0.13; 0.41)	0.315
NH						
Regular care unit – ref	0		0		0	
Special care unit	0.24 (-0.02; 0.49)	0.070	0.08 (-0.19; 0.35)	0.555	-0.64 (-0.91; -0.37)	** < 0.001**

Bold values shown statistically significant result with a *p*-value less than 0.05

Abbreviations: *CI* Confidence interval, *CDR-SoB* Clinical Dementia Rating–Sum of Boxes, *GMHR* General Medical Health Rating, *MOBID-2* Mobilization-Observation-Behavior-Intensity-Dementia-2, *NH* Nursing Home, *NPI-NH* Neuropsychiatric Inventory Nursing Home version, *PSMS* Physical Self- Maintenance Scale, *RC* Regression coefficient, *QUALID* Quality of Life of Late Stage Dementia

^a^Results of linear mixed model analyses. All analyses adjusted for cluster effect within NH; Only cases with no missing values on adjustment variables are included in the analyses

QUALID well-being dimension includes: smiles, enjoys eating, enjoys social interaction and enjoys touching/being touched, QUALID tension dimension includes: physically uncomfortable, verbalization suggests discomfort, irritable and appears calm, QUALID well-being dimension includes: smiles, enjoys eating, enjoys social interaction and enjoys touching/being touched

*N* = 823 (A_1_) + 519 (A_2_) + 299 (A_3_) = 1641

^b^NPI-NH Agitation sub-syndrome: agitation/aggression, disinhibition and irritability; NPI-NH Affective sub-syndrome: depression and anxiety; NPI-NH Psychosis sub-syndrome: delusions and hallucination

^c^Opioids N02A

^d^Paracetamol N02B E01, N02A J06, and N02A J13

In the same adjusted analyses, a higher CDR-SoB score (more severe dementia) was associated with higher QUALID total score and higher sadness and tension dimension scores (poorer QoL) when assessed simultaneously. Prescription of opioids was associated with higher QUALID scores (total and tension dimension), and prescription of paracetamol was associated with higher QUALID scores (total and sadness and tension dimensions), when the factors were assessed simultaneously with the outcome.

Furthermore, in the same analyses, the following factors were associated with higher total QUALID scores or higher QUALID dimension scores when assessed simultaneously: poor physical health (GMHR) (total and well-being dimension); poorer P-ADL (higher PSMS score) (total and tension and well-being dimensions); higher NPI agitation subsyndrome score (total and tension dimension); higher NPI affective subsyndrome score (total and the three dimensions); higher NPI psychosis subsyndrome score (tension dimension); higher NPI apathy score (total and sadness and well-being dimensions); and use of two or more psychotropic drugs (total and sadness dimension). In these analyses, the baseline factors associated with higher QUALID dimension scores at the three assessments were female gender (sadness dimension) and being in a regular care unit (well-being dimension).

## Discussion

In this sample of NH residents with dementia assessed at admission and at 12- and 24-month follow-up assessments, those being prescribed analgesics had a higher pain intensity (higher MOBID-2 overall score and scores of single areas of pain) compared to those not being prescribed analgesics. No trend in QoL over time was found in the adjusted analyses. In the adjusted analyses, being prescribed opioids and/or paracetamol or having a higher pain intensity was associated with poorer overall QoL and two of the three dimensions of QoL (i.e., sadness and tension). Furthermore, in the same analyses, more severe dementia, poor physical health, poorer P-ADL, higher NPI agitation and affective sub-syndrome scores, higher NPI apathy score, and use of psychotropic drugs were associated with poorer overall QoL and some—but not all—dimensions of QoL. Female gender was associated with higher sadness dimension score, and residing in a regular care unit at baseline was associated with lower well-being score.

The mean MOBID-2 overall intensity score in the present study did not vary much between the assessments, but the number of participants declined over time. A previous cross-sectional Norwegian study of NH residents with dementia with varying length of stay at inclusion and a somewhat higher mean age (86.6 years) reported a mean MOBID-2 score of 2.5, which is quite similar to our study result [19]. Furthermore, also in line with our findings, the cross-sectional NH study by van Dam et al. [19] reported that overall MOBID-2 score was significantly higher in those prescribed analgesics than in those not prescribed. The present study adds to the previous findings with its longitudinal design and finding that both the mean MOBID-2 overall score and single MOBID-2 items scores were significantly higher in residents being prescribed either opioids, paracetamol, or both, compared to residents not prescribed analgesics not only at the baseline (A_1_) but also at the follow-up assessments. Our study cannot answer whether the higher MOBID-2 score in those prescribed analgesics than those not prescribed analgesics could be due to pain treatments that are inefficient. Pain treatment in older persons and NH residents with dementia is demanding, complex, and complicated [34, 35, 38]. However, using pain assessment before regular medication reviews may give clinicians a tool with which to evaluate the analgesics prescribed and to minimize the risk of both under- and over-prescription of analgesics [19, 76–79]. Such medical reviews may also contribute to reducing side effects of pain medication as much as possible and to facilitating the best possible QoL in persons with advanced dementia [19].

The mean QUALID score at baseline was almost 20; at the 24-month assessment, the mean score had increased to 22, which indicates poorer QoL over time. In the unadjusted linear mixed model, there was a significant increase in QUALID score (i.e., poorer QoL) over time. However, in the adjusted analysis, no such trend was present, neither in overall QoL nor in the dimensions of QoL; rather, factors indicating pain, health conditions, and prescribed medical treatment of analgesics and psychotropic drugs were associated with overall QoL and the three sub-dimensions of QoL.

In the initial analyses, QoL was found to be significantly lower (higher QUALID total and dimension scores) in those prescribed analgesics than in those not prescribed analgesics at admission and at the first follow-up, but not at the second follow-up. We speculate that the lack of differences after two years may be due to differences in the dropout rates between those prescribed analgesics (43% dropout for different reasons) and those not prescribed analgesics (80% dropout for different reasons) at the final follow-up. It is possible that those dropping out in the group with no prescribed analgesics had worst QoL and in this way contributed to the bias towards falsely improved QoL, which is also supported by descriptive numbers. In the adjusted linear mixed models for QoL where the covariates were assessed simultaneously with outcome, we found that prescription of both opioids and paracetamol, independently of each other, were associated with poorer overall QoL and higher tension, but not with well-being, and prescription of paracetamol was associated with higher sadness. We do not have a firm explanation for these findings, but it cannot be ruled out that the use of analgesics has some side effects that are relevant to QoL. However, van Dam et al. cross-sectionally explored factors associated with QoL in NH residents with dementia, independent of their length of stay prior to the study inclusion, and did not find prescription of opioids or paracetamol to be associated with QoL [19]. The divergence in findings may partly be explained by the differences in methodology and in definition of QoL [47, 48, 80] and, consequently, the scale used (QUALID [45] versus QUALIDEM [81], respectively), but it may also be explained by the fact that the association between paracetamol and QoL was not adjusted for the severity of pain [19].

In the present study, we explored the association between severity of pain and QoL, and in adjusted analyses higher pain severity was associated with higher overall QoL score and higher scores in the QoL dimensions of sadness and tension in NH residents with dementia. The link between pain and overall QoL was expected, even though a systematic review paper from 2013 [45] only found one cross-sectional study exploring and reporting an association between pain and QoL in NH residents with dementia [43]. Newer studies have reported pain to be associated with poorer QoL in NH residents [40] and NH residents with dementia [20, 41, 42]; however, these previous studies were relatively small and cross-sectional, and residents were included independent of their length of stay prior to the assessment.

The well-being dimension of QUALID—the one QoL dimension not associated with pain severity—includes items such as enjoying social interaction and touching/being touched by others. We do not have a firm explanation for this finding, but it may be that social-related aspects of QoL are less affected by severity of pain than physical (tension dimension) and emotional (sadness dimension) related aspects of QoL.

### Strengths and limitations

A major strength of this study is its methodology—primarily, its use of well-known, internationally recognized scales for assessing QoL [53] and pain (MOBID-2) [23, 59]; its use of a measure of cognitive functioning; and the broad experience of the research institution with conducting such studies [8, 60]. The large sample size allowed us to adjust for several factors known to be linked to QoL in NH residents with dementia, including physical health, activities of daily living, neuropsychiatric symptoms, prescription of psychotropic drugs, and demographics, which limited the risk of confounding. Furthermore, the study was conducted at admission to a NH, with annual follow-ups for two years. This made it possible to investigate whether there was a trend in QoL improving or declining over time spent in the NH.

Several limitations must also be mentioned. Firstly, the information regarding pain treatment was restricted: we did not have information about the systematic use of non-pharmacological treatment(s). Other studies have stated that older adults with chronic pain may benefit from non-pharmacological treatment, such as cognitive behavioral therapy, exercise, massage, music therapy, and reflexology [34, 82–84] for pain relief; thus, this may be a co-variate for QoL in NH residents with dementia. The information on analgesics in the present study did not include information about frequency and dosage of prescribed opioids and/or paracetamol, whether analgesics prescribed were actually taken, information regarding analgesics prescribed pro re nata (P.R.N.), non-steroidal anti-inflammatory drugs (NSAIDs), polypharmacy, and the effect of the analgesics prescribed. Information was not collected on the prescribing and deprescribing procedures, nor on the collaboration between the healthcare professionals observing pain location, intensity, and relief, and the physician prescribing, adjusting, and deprescribing analgesics, which may affect pain treatment. Such information could have contributed to a more nuanced understanding of the relevance of pain treatment to QoL in NH residents with dementia.

Secondly, cancer and musculoskeletal disorders are diagnoses that are commonly found in older adults and are related to pain [35], but information about these diseases was missing in this study. Thus, this research study could not explore the associations of these diagnoses or other comorbidities related to pain with the outcome, and with prescription and persistent prescription of analgesics. However, the present study included information about general physical health in the analysis and found that poor general physical health was associated with the prescription of opioids when assessed simultaneously. We cannot guarantee that a reverse association is not present—i.e., that opioids contribute to reduced physical health.

Thirdly, the present study used only one measurement to assess QoL (the QUALID scale) in NH residents with dementia. This was chosen because we emphasized psychological well-being and activities of significance to capture important aspects of QoL in people with dementia. However, we could have broadened the scope of the study by including two dementia-specific QoL inventories, as has been done in other studies [39].

Fourthly, a large number of NH residents (and thus potential participants) were excluded for various reasons other than not having dementia and/or a life expectancy shorter than six weeks. Furthermore, some were excluded due to a significant amount of missing information, which may limit the study’s validity. Lastly, it is a methodological weakness to use a convenience sample instead of a random selected sample of residents newly admitted to a NH. In the present study, data collection was performed in some but not in all NHs of Norway’s counties and not randomly selected. Consequently, the sample is not necessarily representative of older adults with dementia admitted to NHs in Norway, limiting the transferability, and caution should be taken in generalizing the study results.

### Clinical implications

Since this longitudinal study has found pain associated with poorer QoL in NH residents, confirming the results from previous cross-sectional studies [20, 40–43], pain should be an important indicator for quality of care [85–87]. Assessing pain in a reliable way in NH residents is essential to determining and facilitating non-pharmacological and pharmacological pain treatment. Although some countries (the USA, Canada, and Iceland) require NHs to regularly assess pain in all residents using a standardized inventory [85, 87, 88], this is not required in Norway, neither at admission [89] nor thereafter. Regular, systematic assessment of pain and re-assessment after prescription of pain medication should be considered mandatory by administrators and authorities dealing with healthcare service regulations and quality of NH care; this should be followed up by strategies to improve healthcare professionals’ competence and confidence in assessing and interpreting signs of pain in people with dementia, such as noises, facial expressions, and defense-related body movements. This is also important, since pain in NH residents with dementia may be mistaken as neuropsychiatric symptoms [26], and untreated or under-treated pain in people with dementia may trigger neuropsychiatric symptoms [27, 90], which is also associated with poorer QoL.

Non-pharmacological pain management programs are the first line of treatment choice, and they may be administered in combination with analgesics when needed [34]. Thus, systematic non-pharmacological pain management programs need to be developed and introduced in NHs [91].

Since pain treatment in NH residents and particularly residents with dementia is complicated and demanding [34, 35, 38], it is important that healthcare professionals prioritize and improve the identification, monitoring, and treatment of pain [92]. Regular, structured medication reviews to assess use, efficacy, and side effects of prescribed analgesics are one way to promote adequate pain treatment [93], which is mandatory for Norwegian NHs. However, the finding that being prescribed analgesics was associated with poorer QoL may necessitate that pain treatment in NH residents with dementia is given broader attention by healthcare professionals. Interdisciplinary awareness of challenges related to pain [94] and collaboration between nurses, physicians, and pharmacists are essential to effectively assessing and treating pain, to evaluating the effect of pain treatment, and to identifying potential side effects [34]. The application of criteria-based screening tools such as the STOPP/START criteria (Screening Tool of Older Person’s Prescriptions/Screening Tools to Alert Doctors to Right Treatment) [78] and the Norwegian General Practice–Nursing Home criteria (NORGEP-NH) [77] may contribute to appropriate prescription and deprescription of analgesics to NH residents with dementia. Future studies could explore healthcare professionals’ awareness of challenges related to pain and the approaches for assessing pain as well as for treating and evaluating the effects of pharmacological and non-pharmacological pain treatment.

## Conclusion

The present longitudinal observational study of NH residents with dementia found that higher pain intensity and being prescribed analgesics for regular use were associated with poorer QoL when adjusted for socio-demographics, other health conditions, and the use of psychotropic drugs. There was no trend for poorer QoL over time.

This requires the close examination of healthcare professionals and administrators in NHs (and, preferably, national healthcare authorities) into strategies that reduce pain intensity while simultaneously avoiding the negative side effects of pain treatment that hamper the QoL of NH residents with dementia.

### Supplementary Information

**Additional file 1: Supplemental table 1.  **Unadjusted results of linear mixed model assessing factors associated with quality of life, QUALID sadness, tension, and well-being 1.

## Data Availability

The datasets generated and/or analyzed for this study are available to researchers in cooperation with the data owners, the Research Centre for Age-Related Functional Decline and Disease, Innlandet Hospital Trust. Information is available at the following web page: https.//www.sykehuset-innlandet/avdelinger/alderspsykiatrisk-forskningssenter.

## Abbreviations

A_1_: Assessment 1 at baseline
A_2_: Assessment 2 at first follow-up, 12 months
A_3_: Assessment 3 at second follow-up, 24 months
CI: Confidence interval
CDR: Clinical Dementia Rating
CDR-SoB: Clinical Dementia Rating–Sum of Boxes
GMHR: General Medical Health Rating
MOBID-2: Mobilization-Observation-Behavior-Intensity-Dementia-2
N: Number
NH: Nursing home
NPI-NH: Neuropsychiatric Inventory Nursing Home version
NPS: Neuropsychiatric symptoms
P: Statistical significance value
P-ADL: Personal activities of daily living
PSMS: Physical Self-Maintenance Scale
PTD: Psychotropic drug
QUALID: Quality of Life of Late Stage Dementia
QoL: Quality of life
RC: Regression coefficient
SD: Standard deviation
