# Supplementary material for: Pain and quality of life in nursing home residents with dementia after admission – a longitudinal study
Source: BMC Health Serv Res. 2023 Sep 27;23:1032. doi: 10.1186/s12913-023-10041-5 (PMC10537464; doi:10.1186/s12913-023-10041-5)
Supplement: Supplementary file 1 — Additional file 1: Supplemental table 1. Unadjusted results of linear mixed model assessing factors associated with quality of life, QUALID sadness, tension, and well-being 1. [file 12913_2023_10041_MOESM1_ESM.docx]

**Supplemental table 1** Unadjusted results of linear mixed model assessing factors associated with quality of life, **QUALID sadness, tension, and well-being** ^1^.

|  | **QUALID sadness**  Unadjusted models | | | | **QUALID tension**  Unadjusted models | | | **QUALID well-being**  Unadjusted models | | |  |
| --- | --- | --- | --- | --- | --- | --- | --- | --- | --- | --- | --- |
|  | RC (95% CI) | | p-value | | RC (95% CI) | p-value | | RC (95% CI) | | p-value |  |
| Time (months)  0  12  24 | 0  0.30 (0.05; 0.55)  0.43 (0.12; 0.74) | | **0.019**  **0.007** | | 0  0.52 (0.20; 0.83)  1.12 (0.73; 1.51) | **0.001**  **<0.001** | | 0  0.43 (0.23; 0.66)  0.90 (0.64; 1.17) | **<0.001**  **<0.001** | | |
| *Assessed simultaneously with outcome* | | | | | | | | | | | |
| MOBID-2  CDR-SoB  GMHR  Poor/Fairly poor– ref.  Good/Fairly good  PSMS  NPI-NH sub-syndrome^2^  Agitation  Affective  Psychosis  Apathy  Psychotropic drugs (number)  0 – ref.  1  2  3+  Analgesics  Opioids^3^  Paracetamol^4^  Civil status  Unmarried/no partner – ref.  Married/partner | | 0.06 (0.04; 0.07)  0.16 (0.12; 0.21)  0  -0.49 (-0.76; -0.21)  0.10 (0.07; 0.13)  0.11 (0.10; 0.13)  0.33 (0.31; 0.35)  0.19 (0.16; 0.23)  0.23 (0.17; 0.28)  0  0.49 (0.17; 0.81)  1.14 (0.75; 1.52)  2.11 (1.59; 2.62)  0.58 (0.24; 0.93)  0.57 (0.29; 0.85)  0  0.41 (0.06; 0.75) | | **<0.001**  **<0.001**  **<0.001**  **<0.001**  **<0.001**  **<0.001**  **<0.001**  **<0.001**  **0.003**  **<0.001**  **<0.001**  **0.001**  **<0.001**  **0.023** | 0.09 (0.07; 0.10)  0.32 (0.27; 0.37)  0  -0.86 (-1.20; -0.51)  0.21 (0.17; 0.25)  0.30 (0.28; 0.31)  0.38 (0.35; 0.41)  0.34 (0.31; 0.38)  0.22 (0.15; 0.29)  0  0.42 (0.02; 0.83)  1.29 (0.81; 1.78)  2.48 (1.83; 3.14)  1.12 (0.69; 1.56)  0.96 (0.61; 1.32)  0  1.00 (0.56; 1.44) | **<0.001**  **<0.001**  **<0.001**  **<0.001**  **<0.001**  **<0.001**  **<0.001**  **<0.001**  **0.040**  **<0.001**  **<0.001**  **<0.001**  **<0.001**  **<0.001** | | 0.03 (0.02; 0.04)  0.16 (0.13; 0.20)  0  -0.65 (-0.89; -0.42)  0.17 (0.15; 0.20)  0.06 (0.05; 0.08)  0.10 (0.08; 0.13)  0.07 (0.04; 0.10)  0.34 (0.29; 0.38)  0  0.15 (-0.13; 0.43)  0.41 (0.07; 0.75)  0.80 (0.34; 1.26)  0.47 (0.17; 0.76)  0.35 (0.11; 0.59)  0  0.37 (0.07; 0.68) | **<0.001**  **<0.001**  **<0.001**  **<0.001**  **<0.001**  **<0.001**  **<0.001**  **<0.001**  0.299  **0.018**  **0.001**  **0.002**  **0.004**  **0.017** | | |
| *Assessed at baseline* | | | | | | | | | | | |
| Age  Gender  Females – ref.  Males  NH  Regular care unit – ref.  Special care unit | -0.02 (-0.04; -0.001)  0  -0.59 (-0.93; -0.25)  0  0.54 (0.21; 0.88) | | | **0.042**  **0.001**  **0.001** | -0.04 (-0.07; -0.01)  0  0.08 (-0.36; 0.52)  0  0.72 (0.30; 1.15) | | **0.006**  0.733  **0.001** | -0.007 (-0.03; 0.01)  0  0.23 (-0.08; 0.53)  0  -0.62 (-0.91; -0.33) | 0.504  0.141  **<0.001** | | |

Note: Bold values shown statistically significant result with a p-value less than 0.05.

Abbreviations: CI = confidence interval; CDR-SoB = Clinical Dementia Rating – Sum of Boxes; GMHR = General Medical Health Rating; MOBID-2 = Mobilization-Observation-Behavior-Intensity-Dementia-2; NH = Nursing Home; NPI-NH, Neuropsychiatric Inventory Nursing Home version; PSMS = Physical Self- Maintenance Scale; PTD = Psychotropic drug, RC = Regression coefficient, QUALID*=* Quality of Life of Late Stage Dementia

^1^Results of linear mixed model analyses. All analyses adjusted for cluster effect within NH; Only cases with no missing values on adjustment variables are included in the analyses, QUALID well-being dimension includes: smiles, enjoys eating, enjoys social interaction and enjoys touching/being touched N = 823 (A_1_) + 519 (A_2_) + 299 (A_3_) = 1641
^2^NPI-NH Agitation sub-syndrome: agitation/aggression, disinhibition and irritability; NPI-NH Affective sub-syndrome: depression and anxiety; NPI-NH Psychosis sub-syndrome: delusions and hallucination
^3^Opioids = N02A
^4^Paracetamol = N02B E01, N02A J06, and N02A J13.
